# Spatial distribution and associated factors of dropout from health facility delivery after antenatal booking in Ethiopia: a multi-level analysis

**DOI:** 10.1186/s12905-023-02229-y

**Published:** 2023-02-23

**Authors:** Melaku Yalew, Asiressie Molla, Getahun Gebre Bogale, Tesfaye Birhane, Mastewal Arefaynie, Yitayish Damtie, Bereket Kefale, Bezawit Adane

**Affiliations:** 1 Department of Epidemiology and Biostatistics, School of Public Health, College of Medicine and Health Sciences, Injibara University, Injibara, Ethiopia; 2 Department of Epidemiology and Biostatistics, School of Public Health, College of Medicine and Health Sciences, Wollo University, Dessie, Ethiopia; 3 Department of Health Informatics, School of Public Health, College of Medicine and Health Sciences, Wollo University, Dessie, Ethiopia; 4 Department of Reproductive and Family Health, School of Public Health, College of Medicine and Health Sciences, Wollo University, Dessie, Ethiopia; 5 Department of Reproductive Health, School of Public Health, College of Medicine and Health Sciences, Injibara University, Injibara, Ethiopia

**Keywords:** Dropout, Continnum of care, Health facility delivery, Home delivery, Spatial analysis, Multi-level analysis, Ethiopia

## Abstract

**Background:**

Nowadays, retaining women in the continuum of care throughout the lifecycle: adolescence, pregnancy, childbirth, postpartum, and childhood in reproductive health is one of the recent global concerns. Most of the previous studies focused on individual-level factors and used classical logistic regression. Furthermore, it doesn’t take into account its distribution. Therefore, this study aimed to assess spatial distribution, and associated factors of dropout from health facility delivery after antenatal booking among postpartum women in Ethiopia.

**Method:**

Cross-sectional study by secondary analysis of the Ethiopian Mini Demographic and Health Survey (EMDHS) 2019 dataset was conducted among postpartum women. A total of 2882 women who gave birth 5 years prior to the survey were included. Sampling weight was applied and the analysis was done using STATA version 16. Aeronautical Reconnaissance Coverage Geographic Information System (ArcGIS) 10.8 software was used to map the cluster and attribute of dropout from health facility delivery and Global and local Moran's Index methods were used to assess the extent of clustering. Multi-level (two-level) logistic regression analysis was used and variables with a *P* value less than 0.5 were considered statistical significance. Adjusted odds ratio AOR) with a 95% confidence interval was used to show the strength and direction of the association respectively.

**Results:**

Dropout from health facility delivery after ANC (Antenatal Care) booking in Ethiopia was 35.42%, 95% CI (33.70, 37.19), and it spatially clustered (Moran’s index = 0.51, *P* value < 0.001). From individual-level variables: women who were primary educated [AOR = 0.70, 95% CI (0.49, 0.98)], secondary educated [AOR = 0.38, 95% CI (0.19, 0.73)], lived in the middle [AOR = 0.54, 95% CI (0.29, 0.98)], richer wealth [AOR = 0.37, 95% CI (0.18, 0.78)], richest wealth [AOR = 0.21, 95% CI (0.06, 0.74)], being counseled about pregnancy and childbirth complications [AOR = 0.52, 95% CI (0.34, 0.80)] and women who had four and above ANC visit [AOR = 0.52, 95% CI (0.38, 0.71)] were negatively associated with dropout. Whereas, second birth order [AOR = 2.62, 95% CI (1.40, 4.89)], 3–4th birth order [AOR = 4.92, 95% CI (2.82, 8.60)], above 4th birth order [AOR = 4.77, 95% CI (2.16, 10.53))] were positively associated with dropout. From community-level variables: mothers who lived in Afar [AOR = 2.61, 95% CI (1.08, 6.32)] and Oromia [AOR = 2.63, 95% CI (1.15, 6.02)] were positively associated with dropout from health facility delivery after ANC booking.

**Conclusions:**

Dropout from health facility delivery after ANC booking was high as the government’s effort and its spatial distribution in Ethiopia was clustered. Increased educational status of the mother, having four or more ANC visits, counseled about pregnancy and childbirth complications, and higher household wealth were negatively associated and higher birth order, and living in Oromia and Afar region were positively associated with dropout in Ethiopia**.** Strengthening women’s education, encouraging women to complete ANC visits, being counseled them on pregnancy and childbirth complications, and improving family wealth status will be the recalled intervention areas of the government.

## Background

In the past, no single intervention was enough to bring the expected maternal and child health improvement and the continuum of care has been highlighted as a core programmatic principle to reduce morbidity and mortality [1]. In the continuum of care, there are two dimensions namely the time and place or level dimension. In the former, the continuity of care over time from pre-pregnancy (preconception care) to antenatal, childbirth, and postpartum care for women and newborns, and the latter indicates the integrated service delivery provided by the communities at the lower level to the first level (primary health care unit) to higher health care units through referral system [2, 3].

Although health facility delivery should be continued after ANC booking not retaining this continuum of care becomes a great challenge in public health [4–6]. A high dropout rate was observed sequentially from ANC to institutional delivery and from institutional delivery to postnatal care (PNC) in women of developing countries including Ethiopia [7–11]. Studies conducted in Nepal indicated that 39 to 58% of women dropped from health facility delivery after ANC booking [12, 13]. It becomes worse in Africa as evidenced by a study conducted in 28 African countries as 44% of women were dropout [14]. Similarly, studies conducted in different parts of Ethiopia indicated that dropouts from health facility delivery ranged from 22.5 to 62.2% [15, 16].

Due to this high dropout and other problems, maternal mortality remains unacceptably high and Sub-Saharan Africa alone accounts for a 2/3rd of global maternal mortality [17]. In addition, out of 7.6 million under-five children deaths, 44% occurs during the neonatal period and early half of these deaths occurred in the first 72 h following birth [18]. In 2013 alone, 1 million newborns died on the day they were born, and nearly two million newborns died within the first 7 days after birth [19]. Studies indicated that 14 perinatal deaths/1000 births can be averted by health facility delivery [20]. Dropout increases perinatal death [21–23] and the risk of perineal tear, primary postpartum hemorrhage, puerperal sepsis, birth asphyxia, and neonatal infection [21, 23–25].

The government of Ethiopia strives to reduce maternal and child morbidity and mortality by designing different strategies in collaboration with governmental and non-governmental organizations [26, 27]. For instance, Sustainable Developmental Goals (SDGs) emphasize maternal health to deceases MMR (below 199 per 100,000 live birth [28]. In addition, free maternal and child health services (ANC, delivery, and PNC) can decrease maternal and perinatal mortality by 16.4% and 34% respectively [24].

Though, a dropout from health facility delivery after ANC booking has been addressed in previous studies, most of them were accounting only for individual-level variables and they omit clustering [15, 29–33]. In the classical level of analysis, the assumptions of independency among clustered observations may not hold true. Furthermore, the association at the individual-level may not work at the cluster-level and vice versa. Even the existing studies didn’t take into account the spatial clustering of cases and controls. The factors associated with dropout from health facility delivery after ANC booking are area specific and require more than one level of analysis [16, 34, 35]. So, this study took into account those different levels of analysis and aimed to assess the spatial distribution and associated factors of dropout from health facility delivery after antenatal booking among postpartum women in Ethiopia.

## Methods

### Study area, data source, and study period

The study was conducted in Ethiopia, which is located in Eastern Africa and lies between 3° and 15° North latitudes and 33° and 48° East longitudes. It has nine regions and two administrative cities. It occupies a total area of 1.1 million square kilometers estimated from 4620 m above sea level at Ras Dashen to 148 m below sea level at Dallol Depression. There are topographic-induced climatic variations broadly categorized into three: the “Kolla”, or hot lowlands up to approximately 1500 m above sea level, the “Wayna Degas” which range 1500–2400 m above sea level and the “Dega” or cool temperate highlands 2400 m above sea level. It has a total of 104,957,000 population, of which 36,296,657 were women. There was a total fertility rate of 4.6, an infant mortality rate (per 1000 live births) of 48, and a child mortality rate (per 1000 live births) of 67 in the country. Eritrea in the North, Djibouti in the East, Somalia in the East and Southeast, Kenya in the South, South Sudan, and Sudan in the West are the borders of the country. This study used the Ethiopian Mini Demographic and Health Survey (EMDHS) 2019 dataset: The data were accessed from their URL: www.dhsprogram.com by contacting them through a personal account. The survey took place over 3 months, from March, 2019, to June, 2019.

### Study design and population

A cross-sectional study design using a secondary analysis of 2019 EMDHS was used. All reproductive-age women who gave birth 5 years before the survey and started their first antenatal care in Ethiopia were included. But, women whose place of delivery was not recorded were excluded from the study.

### Sample size determination and sampling procedure

**Fig. 1:**
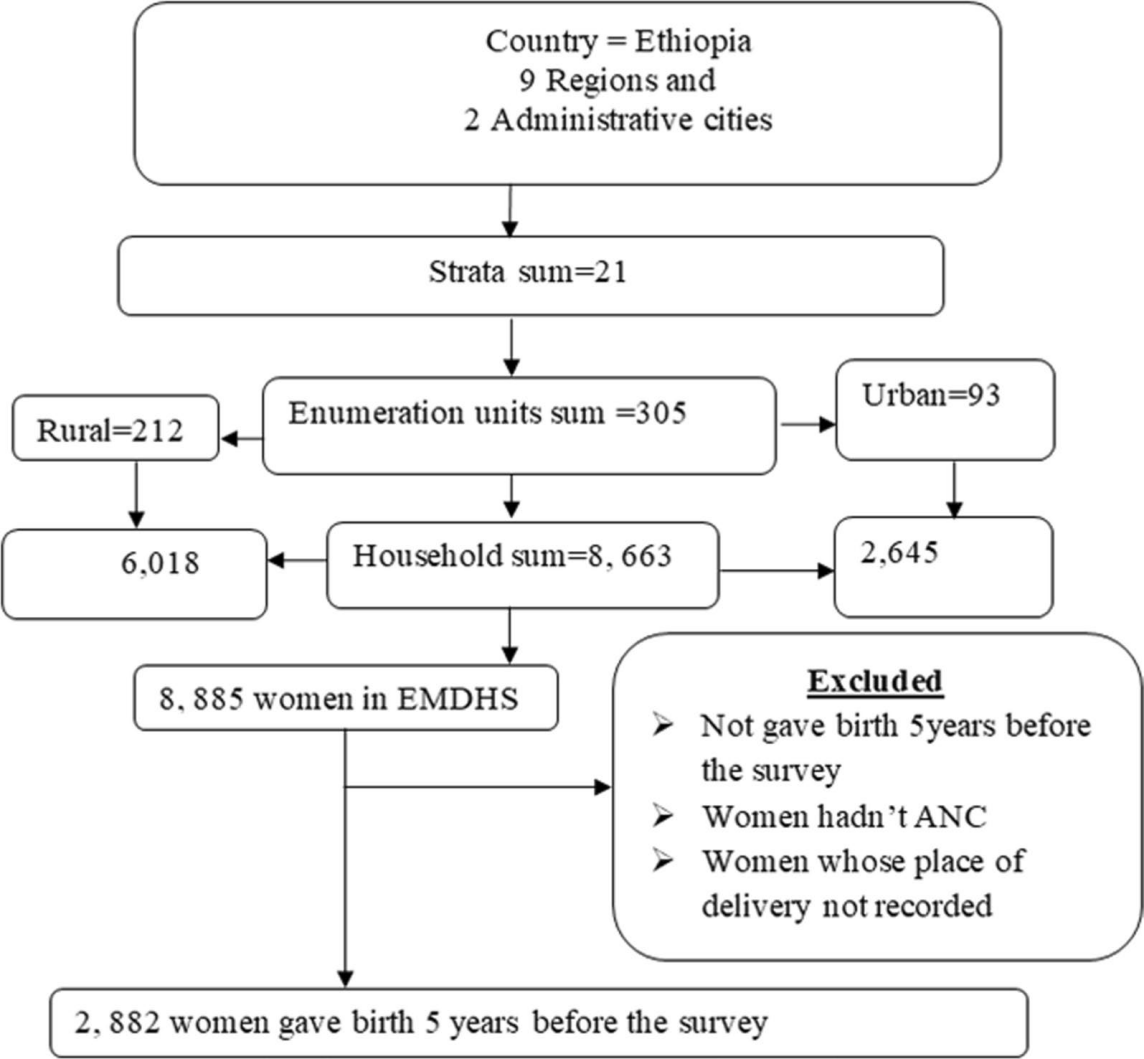
Sampling procedure for spatial distribution and associated factors of dropout from health facility delivery after antenatal booking in Ethiopia

The sampling frame used for the 2019 EMDHS is a frame of all census Enumeration Areas (EAs) created for the upcoming 2019 Ethiopia Population and Housing Census (PHC), which was conducted by the Central Statistical Agency (CSA). Overall, the 2019 EDHS sample contained 305 clusters, and 8885 women aged 15–49 years were interviewed, of which 2882 had given birth in the 5 years before the survey and included from nine geographical regions and two administrative cities of Ethiopia (Fig. 1).

### Variable measurement

The outcome variable for this study is dichotomized as a dropout from health facility delivery (yes/no) which was generated from a constructed EDHS variable. A woman will be coded as 1 (dropout) if she gave birth at home otherwise 0 (not dropout) if she gave birth in a health facility [26]. By aggregating the individual-level variables, community-level variables were generated since EDHS didn’t collect community-level variables directly except residence and region. The aggregates were computed using the proportion of a given variable’s subcategory under a given cluster. Since the aggregate value for all generated variable were not normally distributed. The aggregated value was grouped based on the national median values.

*Community-level of female education* Aggregate respondent level of education is categorized as: Low if less than 50% of the women in the cluster were educated to secondary and above and high if 50 or more percent of women in the cluster were educated to secondary and above [26].

*Community-level of poverty* Aggregate respondent level of wealth is categorized as: Low if less than 50% of the household in the cluster were living below the middle wealth quintile and high if 50 or more percent of the household in the cluster were living below the middle wealth quintile [26].

*Community-level media exposure* Aggregate respondent level of TV/radio availability is categorized as: Low if less than 50% of the household in the cluster had TV/radio and high if 50 or more percent of the household in the cluster had TV/radio [26].

### Data processing and analysis

#### Descriptive and multi-level analysis

Data cleaning was conducted to check for consistency and missing value. Recoding, labeling, and exploratory analysis were performed by using STATA version 16. Descriptive statistics like frequencies and percentages in tables, graphs, and using texts were used to describe the participants. Sample weight was used and multilevel analysis was conducted after checking the existence of significant Intra-cluster Correlation (ICC). Since DHS data are hierarchical, i.e. individuals (level 1) were nested within communities (level 2), and a two-level mixed-effects logistic regression model was fitted to estimate the independent effects of the explanatory variables. The log of the probability of the dropout from health facility delivery after ANC booking was modeled using a two-level multi-level model as follows:

$${\text{Log}}\left[ {\frac{{\Pi {\text{ij}}}}{{1 - \Pi {\text{ij}}}} } \right] =\upbeta _{0} +\upbeta _{1} {\text{X}}_{{{\text{ij}}}} + {\text{B}}_{2} {\text{Z}}_{{{\text{ij}}}} +\upmu _{{\text{j}}} + {\text{e}}_{{{\text{ij}}}}$$

where, i and j are the level 1 (individual) and level 2 (community) units, respectively; X and Z refer to individual and community-level variables, respectively; πij is the probability of dropout from health facility delivery for the ith women in the jth community; the β’s indicates the fixed coefficients. Whereas, β0 is the intercept-the effect on the probability of the dropout from health facility delivery in the absence of influence of predictors; and uj showed the random effect (effect of the community on dropout from health facility delivery for the jth community) and eij showed random errors at the individual levels.

The measures of variation (random effects) were reported using Intra-cluster correlation (ICC), Median Odds Ratio (MOR), and proportional change in variance (PCV). ICC was used to explain cluster variation while MOR is a measure of unexplained cluster heterogeneity. The ICC shows the variation in dropout from health facility delivery after ANC booking for mothers due to community characteristics. The higher the ICC, the more relevant the community characteristics for understanding individual variation. MOR is defined as the median value of the odds ratio between the area at the highest risk and the area at the lowest risk when randomly picking out two areas. The proportional change in variance (PCV) measures the total variation attributed to individual-level factors and community-level factors in the multi-level model.

During analysis first, bi-variable multi-level logistic regression was fitted and variables with p-value less than 0.2 were selected to build the final model. Then the analysis was performed in four steps: Model 1 (empty model or null model/ without explanatory variable), Model 2 (unadjusted/crude model) Model 3 (adjusted for only individual-level factors), and Model 4 (adjusted for both individual and community-level factors). The presence of multi-collinearity was checked among independent variables and there was no multi-collinearity. Log-likelihood ratio test was used to estimate the goodness of fit of the adjusted final model in comparison to the preceding models. A significant log-likelihood ratio test and lower AIC were considered to be the best-fit model.

### Spatial analysis

#### Spatial autocorrelation

Moran's spatial autocorrelation method was computed to assess the extent of clustering in the regions/zones. Moran's I test statistic was computed to test the null hypothesis of no significant clustering in the entire study region/zones. Anselin local Moran’s index was also used to identify a significant neighborhood clustering [36, 37].

- High–high: Positive spatial autocorrelation that indicates high-value clustering.
- Low–low: Positive spatial autocorrelation that indicates clustering of low-value
- Low–high: Negative spatial autocorrelation indicates that low-value rates are adjacent to high value rates.
- High–low: Negative spatial autocorrelation that indicates that high-values are adjacent to low value rates and
- Not significant indicates that there is no spatial autocorrelation.

### Getis OrdGi* statistic (hot spot analysis)

Hotspot statistics was computed to measure how spatial autocorrelation varies over the study location by calculating Gi* statistic for each area. Z-score was used to determine the statistical significance clustering of dropouts from health facility delivery.

### Spatial Satscan statistic and interpolation

Satscan software was used to analyze the purely spatial clusters of dropouts from health facility delivery. A Bernoulli-based model was used and the cluster with the greatest maximum likelihood ratio was considered as the primary cluster. The other statistically significant cluster that didn’t overlap with the primary cluster was identified as secondary clusters, and ranked according to their likelihood ratio test statistic. A spatial interpolation technique was applied to predict the unsampled /unmeasured value of dropout. Spatial interpolation map created by continuous images was produced by interpolating (Kriging interpolation method) dropouts from health facility delivery cases. ArcGIS software was used to map the cluster and attribute of dropout from health facility delivery [38–40].

## Results

### Socio-demographic characteristics of the participants

In the 2019 EMDHS, a total of 9150 households were selected for the sample, of which 8794 were occupied. From the occupied households, 8663 were successfully interviewed yielding a response rate of 98.50%. From the interviewed households, 9, 012 reproductive-aged women were identified for the interview and the interview were completed with 8885 women, yielding a response rate of 98.60%. The median (IQR) age of participants the mother was 28 (± 8) years and the median (IQR) age at first birth was 18 (± 5) years. Only, one hundred fifty-two (5.28%) were educated higher education and above whereas 1262 (43.81%) were not educated. More than two-thirds (70.35%) of the mothers were rural residents and 167 (5.80%) of them were single or never in a union at the time of the survey (Table 1).

**Table 1:** Socio-demographic characteristics of mothers who gave birth 5 years prior to the survey in Ethiopia

Variables	Category	Frequency	Percentage
Age of the mothers in complete years	15–19	153	5.31
	20–24	595	20.63
	25–29	939	32.59
	30–34	591	20.52
	35–39	401	13.91
	40–44	156	5.41
	45–49	47	1.64
Age of the mothers at first birth	Less than 18 years	1150	39.91
	18 and above	1732	60.09
Sex of the child	Male	1540	53.45
	Female	1342	46.55
Marital status	Single or never in union	167	5.8
	Married or living together	2715	94.2
Wealth index	Poorest	396	13.74
	Poorer	575	19.96
	Middle	585	20.3
	Richer	572	19.85
	Richest	754	26.16
Religion	Orthodox	1199	41.61
	Protestant	785	27.24
	Muslim	866	30.06
	Others	31	1.09
Sex of the child	Male	1540	53.45
	Female	1342	46.55

### Obstetric, and household characteristics of the participants

Out of all study participants, 563 (19.52%) mothers were grand multiparous. About one thousand eighty-two (37.54%) mothers started their first ANC in the first trimester and 1671 (57.98%) of them had four and above ANC visits. Of the total participants, nine hundred seventy-one (33.70%) of mothers were living below the middle wealth quintile and 754 (26.16%) belonged to the richest. About, eight hundred forty-nine (29.46%) mothers had above seven family members. More than one in ten households (13.26%) was headed by females (Table 2).

**Table 2:** Obstetric, and household characteristics of the participants in Ethiopia

Variables	Category	Frequency	Percentage
Birth order	First	681	23.65
	Second	610	21.15
	Three to four	741	25.72
	Above forth	849	29.48
Parity	Multipara	2319	80.48
	Grand multipara	563	19.52
Counseled about pregnancy complications	No	1147	39.81
	Yes	1734	60.19
Time of first ANC visit	First trimester	1082	37.54
	Second trimester and above	1800	62.46
ANC complete	No	1211	42.02
	Yes	1671	57.98
Wealth index	Poorest	396	13.74
	Poorer	575	19.96
	Middle	585	20.3
	Richer	572	19.85
	Richest	754	26.16
Family size	1–7	2033	70.54
	Above 7	849	29.46
Sex of household head	Male	2500	86.74
	Female	382	13.26
Household media exposure	No	1685	58.49
	Yes	1196	41.51

### Community characteristics of the participants

In terms of place of residence and region, 2027 (70.35%) were living in a rural area and 1069 (37.11%) were living in Oromia region. Nearly half (48.12%) of mothers lived in low community female education and 1332 (46.23%) were living in a community with high poverty (Table 3).

**Table 3:** Community characteristics of the participants in Ethiopia

*Place of residence*		
Urban	854	29.65
Rural	2027	70.35
*Region*		
Tigray	268	9.3
Afar	32	1.1
Amhara	696	24.15
Oromia	1069	37.11
Somali	63	2.17
Benishangul	39	1.34
SNNP	553	19.19
Gambela	16	0.57
Harari	9	0.3
Addis Ababa	120	4.15
Dire Dawa	17	0.6
*Community media exposure*		
Low	1435	49.81
High	1446	50.19
*Community female education*		
Low	1387	48.12
High	1495	51.88
*Community poverty*		
Low	1549	53.77
High	1332	46.23

### Dropout from health facility delivery after ANC booking

**Fig. 2:**
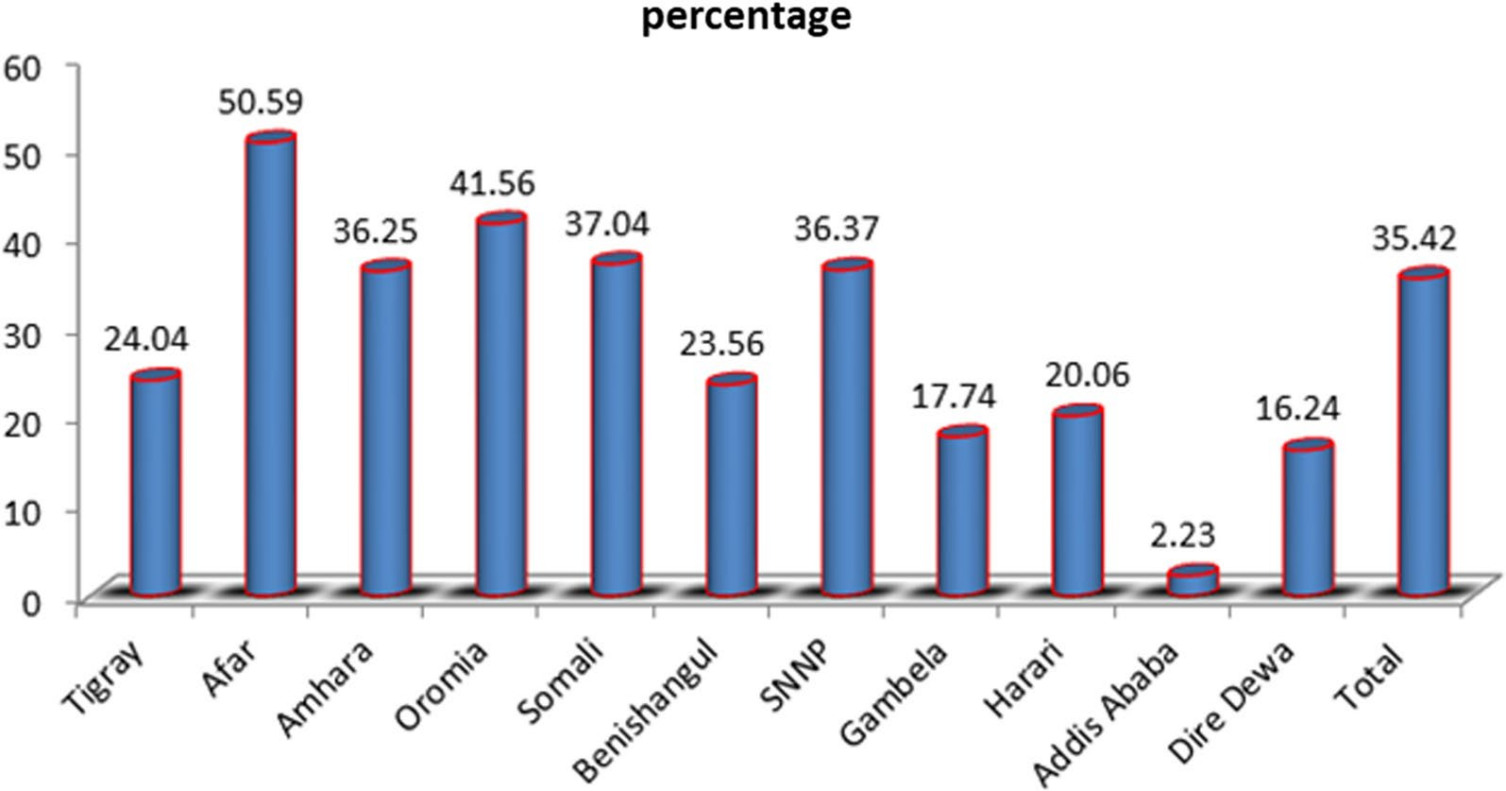
Prevalence of dropout from health facility delivery after ANC booking across different regions of Ethiopia

The overall dropout from health facility delivery after ANC booking among postpartum women in Ethiopia was 35.42%, 95% CI (33.70, 37.19). The lowest (2.23%) and the highest (50.59%) was observed in Addis Ababa and Afar region respectively. The second and the third highest dropout were recorded in Oromia and Somali regions respectively (Fig. 2).

### Spatial distribution of dropout from health facility delivery after ANC booking

#### Global and local indicator spatial autocorrelation

**Fig. 3:**
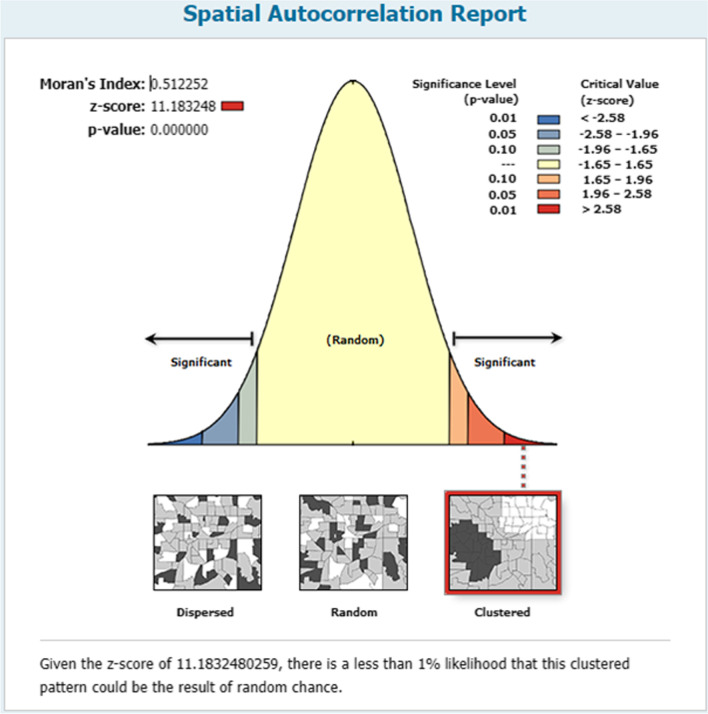
Global spatial auto-correlation (Moran’s I) for dropout from health facility delivery after ANC booking

**Fig. 4:**
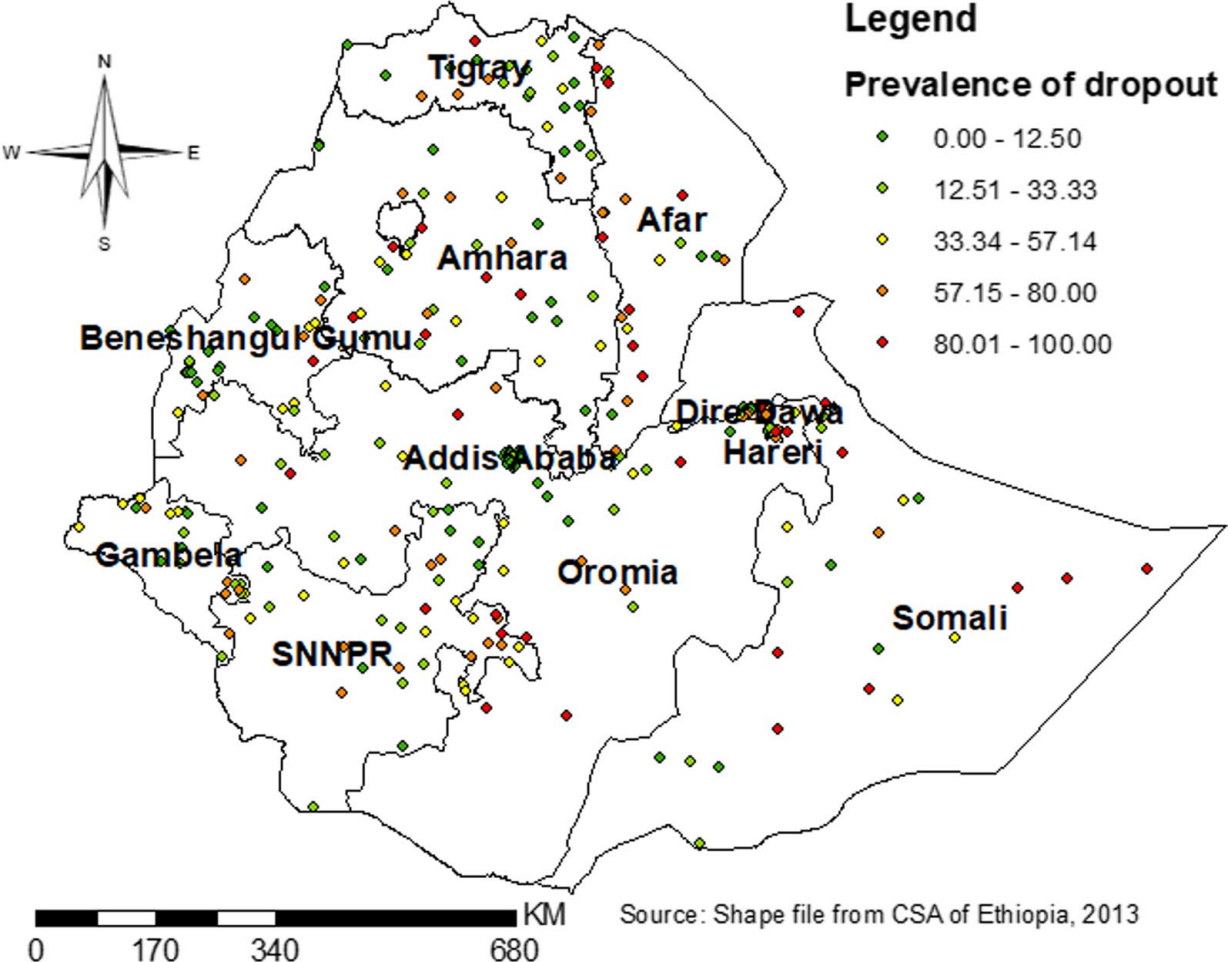
Spatial distribution of dropout from health facility delivery after ANC booking in Ethiopia by different regions

At a regional level, there was spatial variation in dropout from health facility delivery after ANC booking in Ethiopia. Its spatial distribution in Ethiopia was found to be significant (Moran’s index = 0.51, *P* value < 0.0001) (Fig. 3). So, the spatial distribution of dropout from health facility delivery was mapped using 303 clusters. Clusters with a high prevalence of dropout was found in Eastern parts of SNNP, Central and Southwest Amhara, north, south, and West Afar, eastern Somali, and Harari region (Fig. 4).

**Fig. 5:**
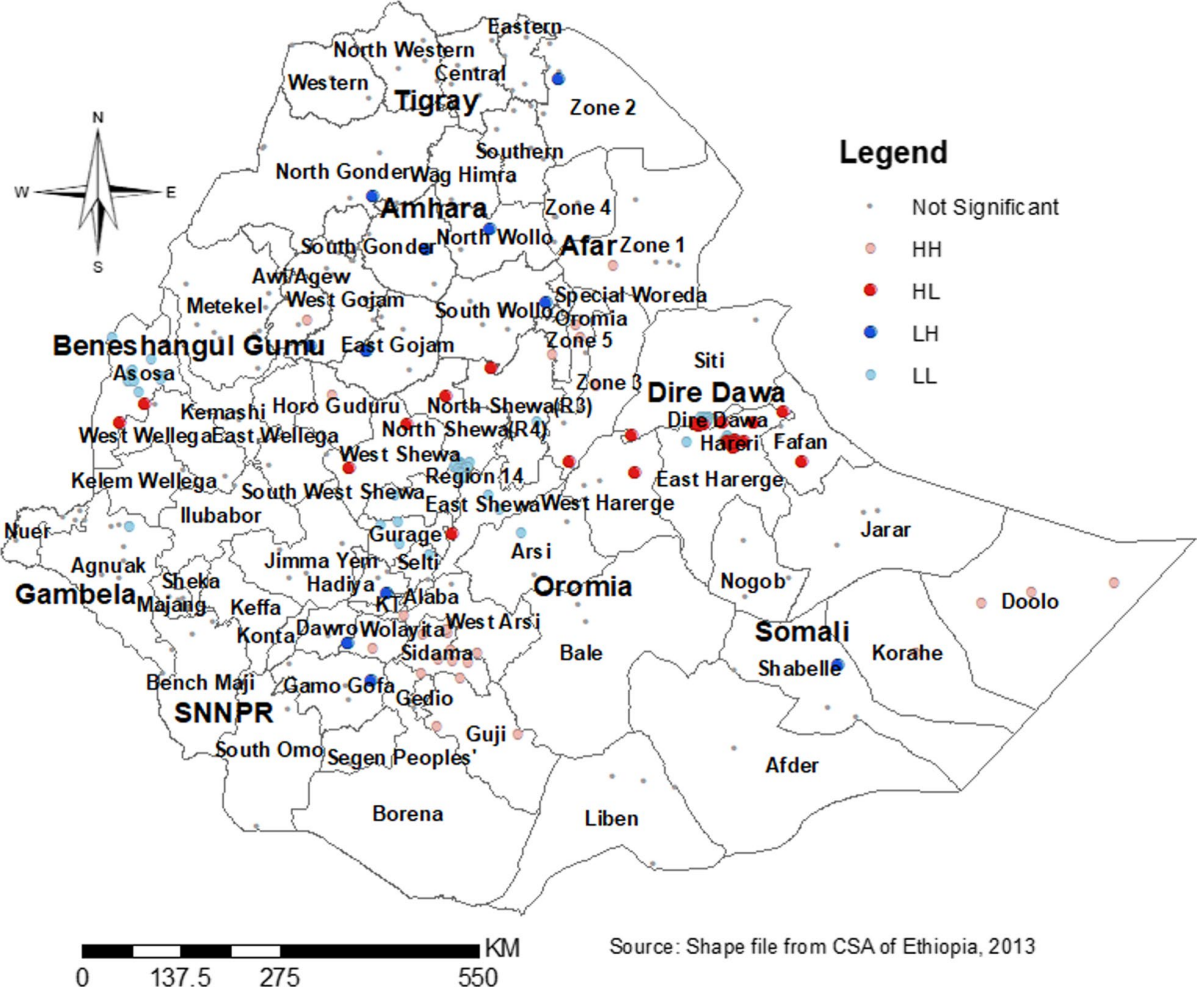
Cluster and outlier analysis (local Moran’s I) of dropout from health facility delivery after ANC booking in Ethiopia by zones

As shown in the figure below, high clustering, and low clustering and spatial outliers (red and blue dots) are very important in identifying significant neighborhood clustering. High clustering (HH) indicate a high prevalence of dropout surrounded by the same characteristics and low clustering (LL) indicate a low prevalence of dropout surrounded by the same characteristics. However, HL and LH indicate a high prevalence of dropout surrounded by a low and a low prevalence of dropout surrounded by a high prevalence respectively. So, these outliers are found Dire Dewa, east Harari, Assosa, north Shewa (HL) and Dawuro Welayta, Gamo-Goffa, north and south Wollo, north and south Gondar (LH) (Fig. 5).

#### Hot spot and Satscan analysis and interpolation

**Fig. 6:**
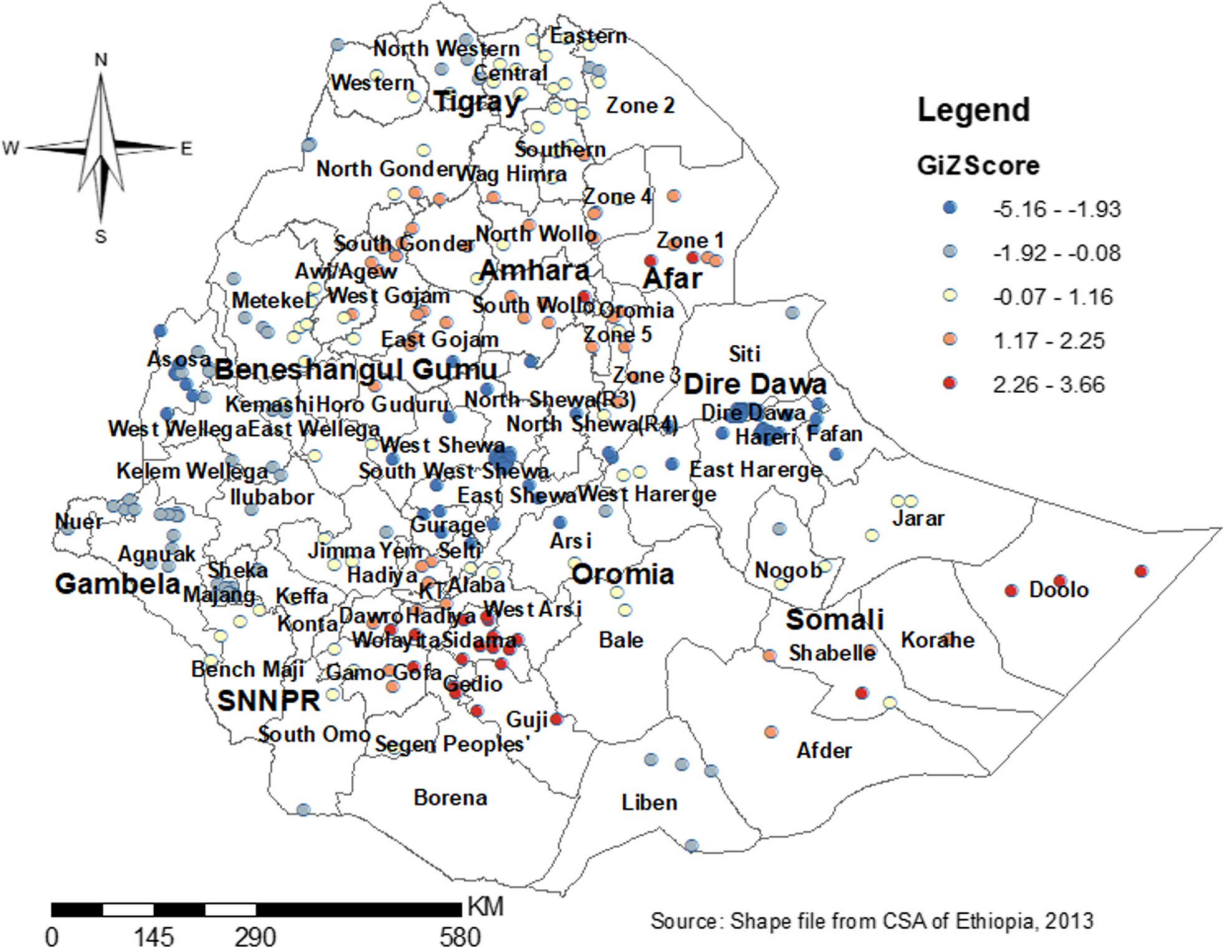
Hot spot analysis for dropout from health facility delivery after ANC booking in Ethiopia by zones

**Fig. 7:**
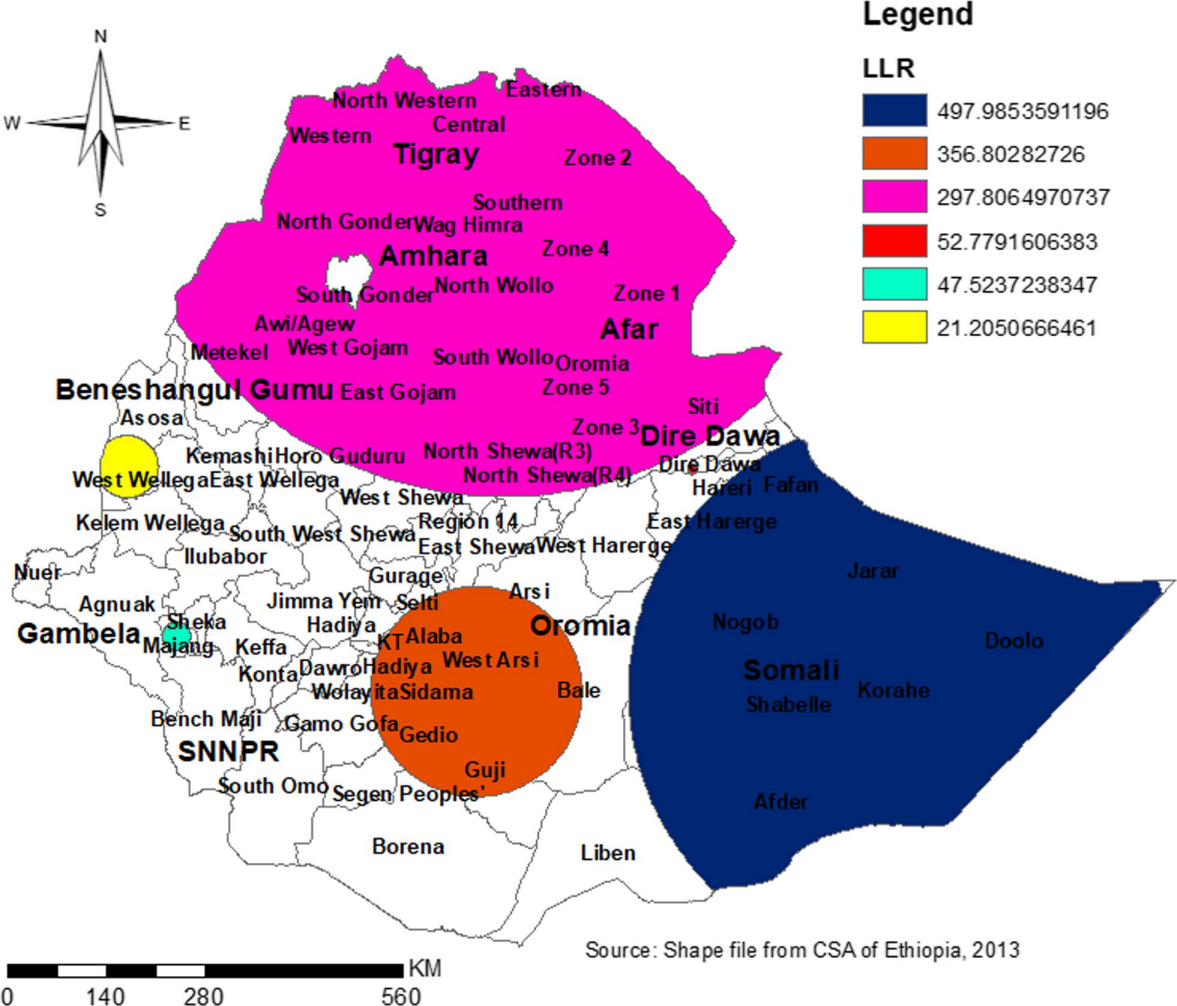
Satscan analysis dropout from health facility delivery after ANC booking in Ethiopia by zones

Gettis-OrdGi* statistics identified the two extreme areas (hot and cold spots). The hot spot indicated the area with a high probability of dropout and the cold spot indicated the area with a low probability of dropout from health facility delivery after ANC booking. So, the most hot spot areas include the dots/clusters in red color which was found in south and north Gondar, central Afar, North and south Wollo, Hadia, Sidama and Geddio zones and the cold spot areas include dots/clusters in blue color which was found Dire Dawa, Harari, Southwest and east Shewa, and Assosa (Fig. 6). Spatial Sat Scan statistics identified primary cluster and secondary clusters. The primary cluster included nearly all parts of Somali region and some parts of Harari and Oromia. But, secondary clusters include: all parts of Tigray and Afar region, major parts of Amhara region, some parts of Oromia, and SNNP regions (Fig. 7) (Table 4).

**Table 4:** Spatial SatScan statistics identified primary cluster and secondary clusters dropout from health facility delivery in Ethiopia

Cluster number	1	2	3	4	5	6
Total population	2501	2000	9404	300	400	200
Number of cases	1616	1266	4233	192	235	115
Prevalence in the area	64.61	63.30	45.00	64.00	58.80	57.50
Relative risk	2.00	1.92	1.48	1.85	1.70	1.65
Log likelihood ratio	497.99	356.80	297.81	52.78	47.52	20.21
*P* value	0.0000001	0.0000001	0.0000001	0.0000001	0.0000001	0.000001
Radius in KM	390.28	150.08	553.25	7.04	19.98	44.25

**Fig. 8:**
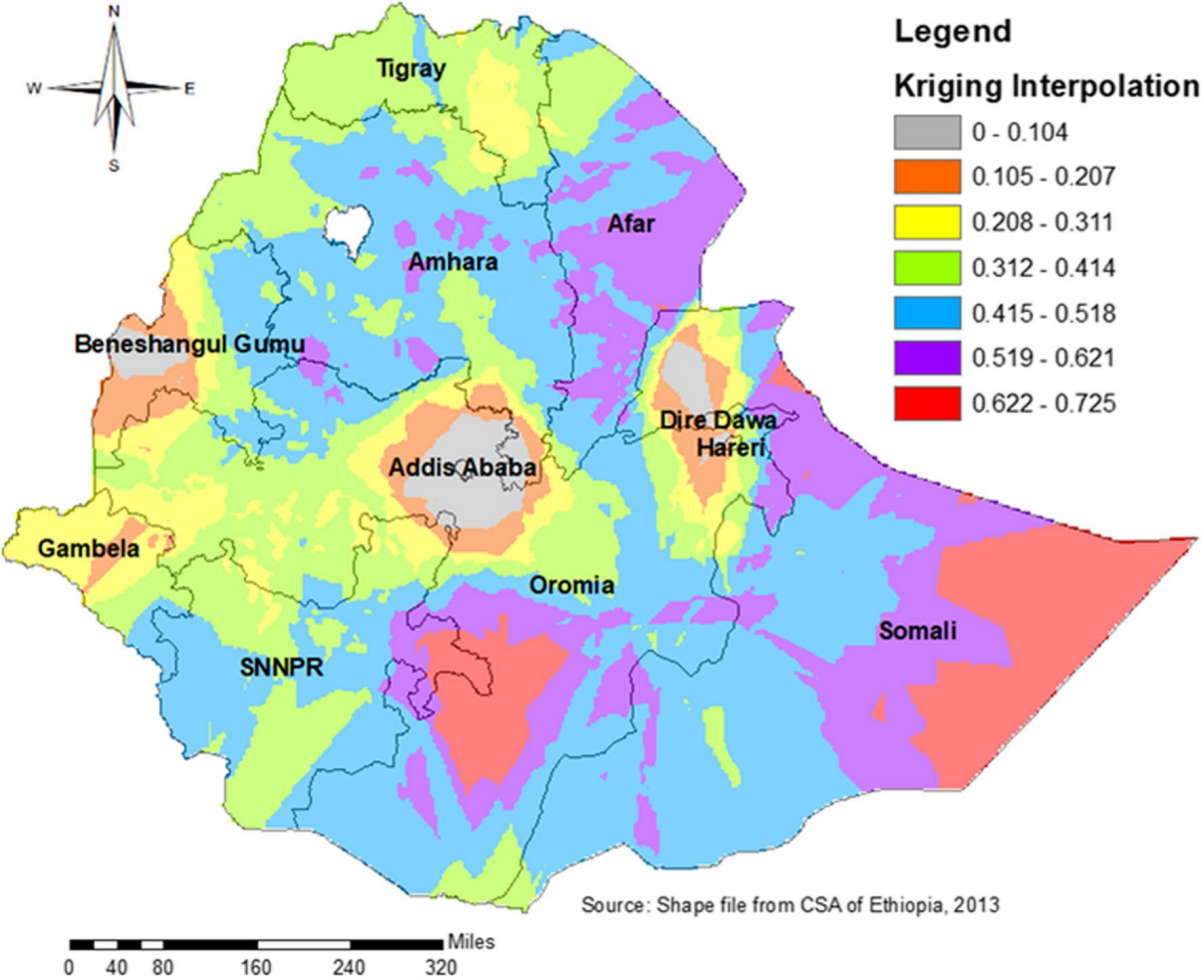
Interpolation for dropout from health facility delivery after ANC booking in Ethiopia by zones

The spatial kriging interpolation analysis predicted the regions which had a high probability of dropout from health facility delivery. Northern and Eastern part of Somali and SNNP region, Southern part of Oromia region, and the majority of Afar were predicted high probability of dropout as compared to other regions. However, the two administrative cities (Addis Ababa and Dire Dewa) and its surroundings were predicated as having less probability of dropout. The red color indicates the predicted high probability areas and the silver color indicates the predicted less probability areas of dropout (Fig. 8).

### Factors associated with dropout from health facility delivery (fixed-effect)

After the adjustment was done on individual and community-level factors (keeping the effect of clustering and other variables constant) six variables were found to be statistically significant. From individual-level variables: educational status of the mother, having four or more ANC visits, counseled about pregnancy and childbirth complications, birth order, and household wealth and from community-level factors only region was significantly associated with dropout from health facility delivery after ANC booking in Ethiopia. The odds of dropout from health facility delivery for those women who were primary educated were 30% less likely as compared to those not educated [AOR = 0.70, 95% CI (0.49, 0.98)]. Similarly, those women who were secondary educated were 62% less likely as compared to those not educated [AOR = 0.38, 95% CI (0.19, 0.73)].

Those mothers who lived in the middle, richer and richest were 46% [AOR = 0.54, 95% CI (0.29, 0.98)], 63% [AOR = 0.37, 95% CI (0.18, 0.78)], and 79% [AOR = 0.21, 95% CI (0.06, 0.74)] less likely to dropout as compared to the poorest respectively.

The analysis also showed that the odds of dropout from health facility delivery for those mothers who were on second, three to fourth and above four birth order were three times [AOR = 2.62, 95% CI (1.40, 4.89)], five times [AOR = 4.92, 95% CI (2.82, 8.60)] and five times [AOR = 4.77, 95% CI (2.16, 10.53))] more likely as compared to the first order respectively. Those women who were counseled about pregnancy and childbirth complications were 48% less likely to dropout as compared to those not counseled [AOR = 0.52, 95% CI (0.34, 0.80)]. Those women who had four and above ANC visits were 48% less likely as compared to those less than four [AOR = 0.52, 95% CI (0.38, 0.71)]. Lastly, the odds of dropout from health facility delivery for those mothers who lived in Afar and Oromia were three times [AOR = 2.61, 95% CI (1.08, 6.32)], three times [AOR = 2.63, 95% CI (1.15, 6.02)] more likely as compared to the Tigray respectively (Table 5).

**Table 5:** Multi-level mixed effect regression for dropout from health facility delivery after ANC booking in Ethiopia

	Model 1	Model 2,	Model 3	Model 4
	ICC = 48.78%	COR (95% CI)	ICC = 36.39%	ICC = 35.89%
			AOR (95% CI)	AOR (95% CI)
*Age of the mother in completed years*				
15–19		1		
20–24		0.98 (0.50, 1.92)	0.87 (0.38, 1.97)	0.84 (0.37, 1.92)
25–29		1.76 (0.90, 3.44)	0.68 (0.24, 1.94)	0.68 (0.24, 1.94
30–34		2.27 (1.13, 4.54)	0.59 (0.21, 1.61)	0.61 (0.22, 1.66)
35–39		1.92 (0.93, 3.95)	0.38 (0.12, 1.20)	0.39 (0.12, 1.27)
40–44		3.05 (1.35, 6.90)	0.68 (0.21, 2.20)	0.68 (0.21, 2.23)
45–49		1.15 (0.31, 4.25)	0.20 (0.04, 1.07)	0.20 (0.04, 1.11)
*Age of the mother at her first birth in complete years*				
Less than 18 years		1		
18 years or above		0.80 (0.58, 1.11)	1.26 (0.88, 1.81)	1.28 (0.89, 1.84)
Educational status of the mother				
Not educated		1		
Primary		0.47 (0.36, 0.61)	0.69 (0.49, 0.97)	**0.70 (0.49, 0.98)**
Secondary		0.15 (0.08, 0.28)	0.37 (0.19, 0.72)	**0.38 (0.19, 0.73)**
Higher		0.13 (0.03, 0.52)	0.62 (0.13, 2.97)	0.69 (0.14, 3.39)
*Wealth index of the household*				
Poorest		1		
Poorer		0.49 (0.28, 0.85)	0.59 (0.33, 1.04)	0.59 (0.33, 1.07)
Middle		0.41 (0.24, 0.69)	0.52 (0.30, 0.92)	**0.54 (0.29, 0.98)**
Richer		0.21 (0.11, 0.40)	0.34 (0.17, 0.69)	**0.37 (0.18, 0.78)**
Richest		0.06 (0.02, 0.16)	0.13 (0.04, 0.39)	**0.21 (0.06, 0.74)**
*Household media exposure*				
No		1		
Yes		0.46 (0.30, 0.72)	0.85 (0.53, 1.36)	0.86 (0.53, 1.39)
*Counseled about pregnancy and childbirth complications*				
No		1		
Yes		0.40 (0.27, 0.59)	0.52 (0.34, 0.81)	**0.52** (**0.34, 0.80)**
*Have four or more ANC visit*				
No		1		
Yes		0.41 (0.29, 0.56)	0.52 (0.38, 0.70)	**0.52** (**0.38, 0.71)**
*Gestational age at their first ANC visit*				
First trimester		1		
Second and above		1.79 (1.28, 2.52)	1.21 (0.80, 1.82)	1.20 (0.80, 1.81)
*Parity*				
Multiparity		1		
Grand multiparity		1.53 (1.07, 2.18)	0.93 (0.53, 1.61)	0.91 (0.52, 1.59)
*Birth order*				
First		1		
Second		2.34 (1.35, 4.05)	2.60 (1.41, 4.80)	**2.62 (1.40, 4.89)**
3–4		3.88 (2.41, 6.27)	5.05 (2.88, 8.86)	**4.92 (2.82, 8.60)**
Five and above		3.94 (2.36, 6.58)	4.81 (2.23, 10.38)	**4.77 (2.16, 10.53)**
*Place of residence*				
Urban		1		
Rural		13.78 (6.81, 27.89)		2.26 (0.92, 5.56)
*Region*				
Tigray		1		
Afar		5.06 (1.63, 15.71)		**2.61** (**1.08, 6.32)**
Amhara		2.06 (0.75, 5.71)		1.86 (0.84, 4.13)
Oromia		3.01 (1.07, 8.49)		**2.63** (**1.15, 6.02)**
Somali		3.91 (1.23, 12.38)		1.06 (0.37, 3.10)
Benishangul		0.87 (0.28, 2.76)		0.56 (0.22, 1.40)
SNNP		2.17 (0.80, 5.87)		1.47 (0.63, 3.43)
Gambela		0.69 (0.21, 2.29)		0.73 (0.28, 1.87)
Harari		0.58 (0.17, 1.98)		1.12 (0.42, 2.96)
Addis Ababa		0.03 (0.01, 0.13)		0.47 (0.12, 1.89)
Dire Dewa		0.43 (0.12, 1.50)		0.81 (0.39, 1.67)
*Community-women education*				
Low		1		
High		0.18 (0.11, 0.31)		1.13 (0.59, 2.14)
*Community poverty status*				
Low		1		
High		7.05 (4.09, 12.14)		1.45 (0.77, 2.70)
*Community media exposure*				
Low		1		
High		0.15 (0.08, 0.26)		0.87 (0.47, 1.61)

Bold means they are statistically significant at alpha value of 5%

### Random effect

The results of random effect analysis indicated that there was a significant correlation between observations taken from the same cluster (ICC = 48.78%). This means about 49% of the variation in dropout from health facility delivery after ANC booking was linked to the community or cluster. The analysis indicated that 41% of the variation of dropout from health facility delivery after ANC booking was explained by the full model. Besides, the MOR confirmed that the dropout from health facility delivery after ANC booking was attributed to community**-**level factors. The MOR for dropout from health facility delivery was 4.57 in the empty model; this indicates that there was variation between communities (clustering) since MOR was 4.57 times higher than the reference (MOR = 1). Despite, the effects of clustering are still statistically significant in the full models, the unexplained community variation in dropout from health facility delivery decreased to MOR of 3.5 when all (individual and community) factors were considered in the model (Table 6).

**Table 6:** Measure of variation for dropout from health facility delivery after ANC booking in Ethiopia

Measures of variation	Model 1 (null model)	Model 2	Model 4
Variance	3.13	1.88	1.84
Proportionate change in variance (PCV)	Reference	39.94	41.21
Median odds ratio (MOR)	4.57	3.54	3.50
Intra-cluster correlation coefficient (ICC)	48.78	36.39	35.89
*Model fitness*			
Log-likelihood	− 1501.41	− 1309.37	− 1294.51
Log-likelihood ratio test	0.0000	0.0083	
Akaike’s An Information Criteria (AIC)	3006.81	2666.75	2665.02
Bayesian Information Criteria (BIC)	3018.75	2895.98	2891.79

## Discussion

This study aimed to describe the spatial distribution and associated factors of dropout from health facility delivery after antenatal booking in Ethiopia. In this study, the prevalence of dropout from health facility delivery after ANC booking was 35.42%, 95% CI (33.70, 37.19) and its distribution was found non-random. Educational status of the mother, having four or more ANC visits, counseled about pregnancy and childbirth complications, birth order, household wealth, and region were significantly associated with dropout in Ethiopia**.** The prevalence of dropout from health facility delivery was in line with a study conducted in Delanta, Ethiopia which was 35.2% [32].

However, the prevalence was lower than studies conducted in Southern Nation Nationalities and Peoples Region (SNNPR) Ethiopia 62.2% [16], Lay Gayint District, Amhara, Northwest Ethiopia 52.7% [29] and two national-level studies in Ethiopia 46.52% [33] and 55.60% [41]. Again the finding of this study was lower than a study conducted in Tanzania (53.81%) [42], Kenya (66.30%) [43], Uganda (48%) [44], Nigeria (38.1%) [34], Zambia (42.70%) [45], Guinea (74%) [46], and rural Gahna (38.1%) [47]. The finding was also lower as compared to a study conducted in 28 African countries (44%) [14] and studies conducted in different parts of Nepal which was ranged from 39 to 58% [12, 13, 48]. The possible reason for this difference may be due to the fact that the first three studies in Ethiopia were conducted in smaller sample sizes and time variation may be attributed to the difference. In addition, context differences in African countries and other developing countries may be attributed to the difference.

On the opposite, the prevalence of dropout from health facility delivery was higher as compared to a study conducted in West Gojjam (31.10%) [49]. It was also higher as compared to a study conducted in Arbaminch, Ethiopia (26.6%) [50]. It was higher as compared to a study conducted in India (10%) [51] and Cambodia (19%) [8]. The possible reason may be context differences in African countries and other developing countries may be attributed to the difference.

Significant clustering was found in the Eastern parts of SNNP, Central and Southwest Amhara, north, south, and West Afar, eastern Somali, and Harari region, and outliers are found in Dire Dewa, east Harari, Assosa, north Shewa, Dawuro Welayta, Gamo-Goffa, north and south Wollo, north and south Gondar. Hotspot areas were found in south and north Gondar, Central Afar, North and south Wollo, Hadia, Sidama, and Geddio zones and the cold spot areas include dots/clusters in blue color which were found in Dire Dawa, Harari, South West and East Shewa, and Assosa. The primary cluster included nearly all parts of the Somali region and some parts of Harari and Oromia. But, secondary clusters include: all parts of the Tigray and Afar regions, major parts of the Amhara region, some parts of Oromia, and SNNP regions. In the spatial kriging interpolation analysis Northern and Eastern parts of the Somali and SNNP region, the Southern part of Oromia region, and the majority of Afar predicted a high probability of dropout as compared to other regions. However, the two administrative cities (Addis Ababa and Dire Dewa) and its surroundings were predicated as having less probability of dropout. Though the government strives to achieve SDG, significant numbers of women dropout from health facility delivery. If the problem persists or gets worse, the expected reduction of maternal and child morbidity and mortality will not be achieved. So, healthcare providers, policymakers, and programmers should invest their resources in women even after ANC booking.

The result of this study indicated that the odds of dropout from health facility delivery after ANC booking were negatively associated with their educational status. The result is congruent with studies undertaken in different parts of Ethiopia [15, 16, 52]. It is also similar to studies done in a multi-county study in 49 and 28 countries including Africa [5, 14]. The result was again supported by a study conducted in Nepal [48]. Studies in Pakistan and Cambodia also showed the same finding [8, 53]. The possible justification for the association may be due to the fact that educated women can easily understand the possible complications of pregnancy and childbirth. On the contrary, uneducated women may perceive as skilled birth attendants and health facility delivery as necessary for women who experienced obstetric complications [54].

The result of this study showed that the odds of dropout from health facility delivery for those mothers who had higher birth order were more likely as compared to the first order. It is congruent with a national-level study conducted in Ethiopia [33]. It is also similar to a study conducted in Nigeria [34] and Cambodia [8]. But, the result is contrary to a study conducted in Tanzania [42]. The possible reason for this difference may be the difference in context and value of children. The reason for the association may be due to poor satisfaction with respect to the previous services or a negative attitude developed from one or others’ experiences. Furthermore, the first birth orders hadn’t experience childbirth and they may fear to dying [55].

Those women who had four and above ANC visits were less likely to dropout as compared to those who had less than four. The finding is similar to a study conducted in Southern Nation Nationality and Peoples Region, Ethiopia [16]. Studies conducted in Cambodia and 28 African countries showed the same evidence [8, 14]. However, it is inconsistent in a study done in Tanzania [42]. The possible reason for this association may be due to the fact that frequent contact between the mother and the healthcare provider may build trust and confidence.

Those women who were counseled about pregnancy and childbirth complications were less likely to dropout as compared to those not counseled. The finding is similar to studies conducted in different parts of Ethiopia [15, 33]. But, it is contrary to a study conducted in 28 African countries [14]. The possible difference for this may be the difference in counseling methods and experiences. The possible reason for the association may be due to the fact that during counseling she may clearly understand as every pregnant woman is at risk.

The final model indicated that the odds of dropout from health facility delivery are negatively associated with mothers’/households’ wealth. The result is similar to a national-level study conducted in Ethiopia [52]. It is also in line with a study conducted in Guinea [34]. Other studies conducted in Pakistan, India and 49 different countries including African countries also showed the same finding [5, 51, 53]. The possible reason for the association may be due to the fact that the higher the wealth they may not worry about the expense during transportation and/or after delivery.

The odds of dropout from health facility delivery for those mothers who lived in Afar and Oromia were more likely as compared to the Tigray. This study is similar to a study conducted in Ethiopia [52]. The possible reason for the association may be due to the fact some parts of the above-mentioned regions are pastoralists and they may have difficulty to staying long periods in one settlement.

This study was conducted by using multilevel logistic regression analysis that can be able to identify the multilevel factors at different levels which will provide important insight to design interventions. The results are representative of the entire Ethiopian population because of appropriate estimation adjustments such as weighting. Moreover, the spatial analysis indicated the most hotspot areas. Despite its strength, the findings of the current study have limitations. Due to the cross-sectional nature of the study design, this paper is limited in its ability to establish a causal relationship between the associated. Due to the use of secondary data, important variables like cultural beliefs in the intra and postpartum period, partners’ factors, and knowledge of danger signs, were not available in the dataset, so these variables were not included in the analysis.

## Conclusions

In this study, the prevalence of dropout from health facility delivery after ANC booking was high as the government’s effort and its spatial distribution was non-random with a positive Moran’s Index. In addition, both individual and community-level factors were significantly associated with dropout from health facility delivery. Increased educational status of the mother, having four or more ANC visits, being counseled about pregnancy and childbirth complications, higher household wealth were negatively associated and higher birth order, living in Oromia and afar region was positively associated with dropout from health facility delivery after ANC booking in Ethiopia. Strengthening women’s education, encouraging women to complete ANC visit, counseling them on pregnancy and childbirth complications, and improving family wealth status will be the recalled intervention areas of the government. Continuous and special attention should be given to women who live in the poorest wealth quintile and those who had higher birth order.

## Data Availability

This study used the 2019 Ethiopia mini Demographic and Health Survey (EDHS) dataset(s). Even if it is open after legal registration, it is restricted to share the dataset to anyone else without consent of DHS. So, it can be easily accessible to everybody interested using a web site: www.measuredhs.com. Then, login at: login at: https://www.dhsprogram.com/data/dataset_admin/login_main.cfm. Data are however available from the corresponding author upon reasonable request and with permission of DHS.

## Abbreviations

ANC: Antenatal care
CSA: Central Statistical Agency
DHS: Demographic and Health Survey
EAs: Enumeration areas
EMDHS: Ethiopian Mini Demographic and Health Survey
